# *Chorta* (Wild Greens) in Central Crete: The Bio-Cultural Heritage of a Hidden and Resilient Ingredient of the Mediterranean Diet

**DOI:** 10.3390/biology11050673

**Published:** 2022-04-27

**Authors:** Andrea Pieroni, Naji Sulaiman, Renata Sõukand

**Affiliations:** 1 University of Gastronomic Sciences, 12042 Pollenzo, Italy; 2 Department of Medical Analysis, Tishk International University, Erbil 4401, Iraq; 3 Department of Crop Sciences and Agroforestry, Faculty of Tropical AgriSciences, Campus Praha-Suchdol, Czech University of Life Sciences Prague, 16500 Prague, Czech Republic; sulaimann@ftz.czu.cz; 4 Department of Environmental Sciences, Informatics and Statistics, Ca’ Foscari University of Venice, 30172 Venezia, Italy; renata.soukand@unive.it

**Keywords:** ethnobotany, wild food plants, Mediterranean diet, food heritage, Greece

## Abstract

**Simple Summary:**

Wild greens (WGs) are thought to have played a crucial role in the post-Neolithic Mediterranean diets of the Near East and the Mediterranean. The current study reports the bio-cultural diversity of WGs (*Chorta*) in Central Crete. Comparison with some Greek historical data of the 19th and 20th centuries shows that WGs have remained resilient and are still crucial in the daily Cretan diet, with an important role played by weedy Asteraceae species. Cross-cultural comparison with the WGs gathered and consumed in the Central and Eastern Mediterranean demonstrates a remarkable diversity of Cretan WGs and important similarities with those consumed in Greek-speaking Cyprus, the Bodrum area of Turkey, coastal Syria, and Southern Italy. The implications of Cretan *Chorta* for both historical studies of the Mediterranean Diet and for promoting WGs-centered food heritage are discussed.

**Abstract:**

An ethnobotanical field study focusing on traditional wild greens (WGs) was carried out in Central Crete, Greece. Through thirty-one semi-structured interviews, a total of fifty-five wild green plants and their culinary uses and linguistic labels were documented; they were mostly consumed boiled (*vrasta*) or fried (*tsigariasta*), as a filling for homemade pies. Comparison with some Greek historical data of the 19th and 20th centuries showed that WGs have remained resilient and are still present in the current Cretan diet. Cross-cultural comparison with the WGs gathered and consumed in other areas of the Central and Eastern Mediterranean demonstrated a remarkable diversity of Cretan WGs and important similarities with those consumed in Greek-speaking Cyprus, the Bodrum area of Turkey, coastal Syria, and Southern Italy. We discussed the cognitive categories linked to *Chorta*, as well as the possible origin of an original “bulk” of post-Neolithic food weeds that could have spread from the Fertile Crescent westwards across the Mediterranean basin over a few millennia. The current study represents a crucial effort to document and preserve the bio-cultural gastronomic heritage of *Chorta* and it is advisable that both biology and history scholars, as well as policy makers, pay needed attention to the WGs of the Cretan and Mediterranean diet.

## 1. Introduction

The Mediterranean diet (MD)—the theorization of which was proposed for the first time in the cross-cultural epidemiological “Seven Countries Study” by the American nutritionist Ancel Benjamin Keys [1]—has been ascribed to “food patterns typical of Crete, much of the rest of Greece, and southern Italy in the early 1960s, where adult life expectancy was among the highest in the world and rates of coronary heart disease, certain cancers, and other diet-related chronic diseases were among the lowest” [2]. The MD was recognized one decade ago as a UNESCO Intangible Cultural Heritage of Humanity [3]. This food system has been the subject of vast biomedical literature with important outcomes in terms of public health (see for example [4,5,6] and references therein), but details on the specific (especially wild) ingredients, traditional food products, and culinary preparations of this food heritage, as well as their underlying historical development, are largely unexplored. We described a few years ago the wild vegetable (WGs-based) portion of the traditional Mediterranean food system as a “hidden MD” [7], mainly for two reasons: (a) archaeological studies cannot help much in providing very robust data on wild leafy vegetables used in the past, since charred plant remains do not primarily include small leaves and leaf rosettes; (b) these ingredients have been neglected by traditional MD bio-nutritional narratives, which have favored cultivated vegetables, because of both the cultural distance between American-centric research and the actual daily MD cultures and the objective lack of in-depth botanical knowledge of many scholars, who have addressed the biology of the MD.

The wild plant portion of the MD, however, has been investigated by a considerable number of ethnobotanical field studies in the Central and Eastern Mediterranean region [8,9,10,11,12,13,14,15,16,17,18,19] during the past two decades; these studies have aimed at documenting the botanical identity and local folk names and uses of foraged species and, to a lesser extent, their folk cognitive features and exact culinary transformations, as well as their nutraceutical properties [20,21,22,23,24,25]. Moreover, a systematic comparison of the foraged plant ingredients of the MD is still lacking, despite the fact that this work could be crucial for understanding the development of Mediterranean rural cuisines. It is in fact accepted that the spread of the use of weedy WGs in the MD originated in Neolithic settlements and that these plant resources often represented both food and medicine [26]. However, still needed in the wild food ethnobotany of the Mediterranean and Near Eastern areas are cross-cultural and diachronic field studies, so as to understand temporal and spatial changes of this crucial bulk of food ingredients. This could be relevant to a series of possible future horizons: (a) food heritage: revitalization of neglected ingredients in a new gastronomic arena and local-food-centered rural ecotourism; (b) community-centered biological diversity management of wild food plants with attached landscapes and environmental education; (c) prehistory and history of Mediterranean food; and (d) further bio-pharmacological and nutritional evaluations of neglected ingredients of the MD and possible further development of health claims for specific neglected WGs.

In the field of wild food ethnobotany, Greece has been a kind of black spot, despite the fact that the coastal part of the country is considered the home of MD studies [2]. To our knowledge, only three economic botanical and ethnobotanical works have been focused on Greek wild greens (*Chorta*) and published in Western European languages: one was conducted in the 19th century by the German botanist Theodor Heinrich Hermann von Heldreich [27], another in 1970 on Corfu Island by the archeologist Augustus Sordinas [28], while a third survey on the uses of ten edible plants in Eastern Crete was published a decade ago, as one of the main outcomes of a PhD research work, published in Greek [29].

We therefore decided to conduct wild food ethnobotanical research on *Chorta* in Crete, which is well known for the widespread popularity of its WGs in the winter and spring folk cuisine [30].

The field work aimed to study *Chorta* in present-day central Crete and to compare the data historically and cross-regionally in the Eastern and Central Mediterranean.

The specific objectives of this research were:

(a) To record local phytonyms and traditional uses of WGs in central Crete;
(b) To compare the gathered data with that collected and reported approximately 100 and 50 years ago in Greece;
(c) To compare the current Cretan data with the surrounding Eastern and Central Mediterranean areas where ethnobotanical studies on WGs have been conducted in the past few decades.

## 2. Materials and Methods

### 2.1. Study Area and Communities

**Figure 1:**
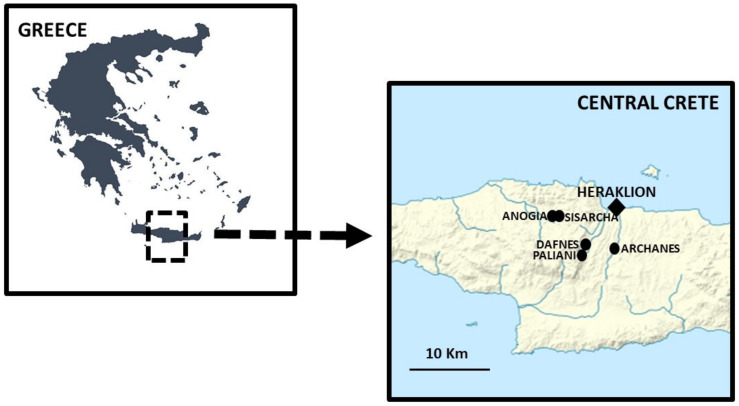
Location of the study area and visited villages.

The field study was carried out in central Crete, Greece, in both the town of Heraklion and a few surrounding rural villages (Figure 1).

**Figure 2:**
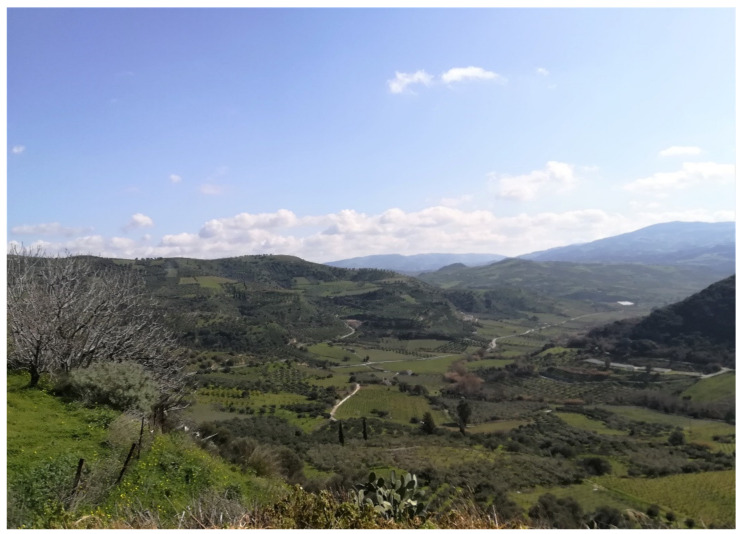
The horticulture-driven landscape in the area of Paliani.

**Figure 3:**
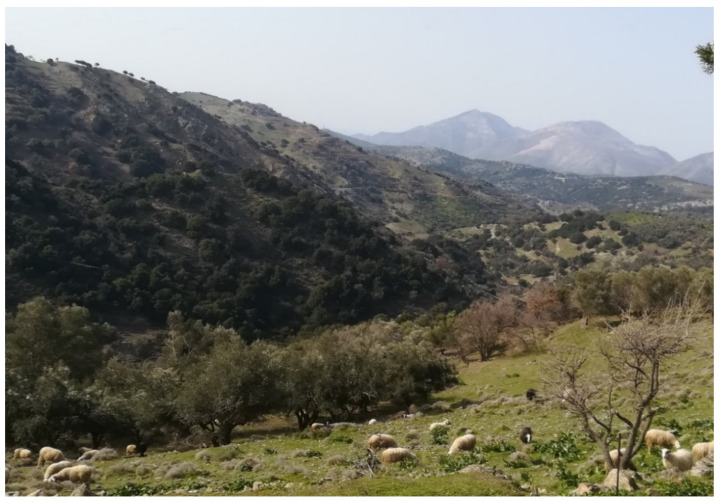
The pastoralist-driven landscape in the rural area of Anogia.

The rural landscape is characterized by typical olive orchards and vineyards at lower altitudes and by shrublands, pastures, and sheep herding at higher altitudes (Figure 2 and Figure 3); the local economy is based on small-scale farming and tourism.

### 2.2. Cretan Biological Diversity

Climatically, the Cretan ecoregion is characterized by a sharp altitudinal gradient; warm and dry low plains have an average annual temperature of about 17–19 °C, with total rainfall of less than 300 mm in the southeastern part of the island, while cold and humid higher elevations have an average annual temperature of about 9–13 °C, with total rainfall of up to 1400 mm [31]. From a geological point of view, Crete’s mountain ranges belong to the Alpine orogenic system, characterized by the predominance of Mesozoic and Tertiary sedimentary rocks, while the karstic landforms are impressive [31].

Cretan Mediterranean forests cover a very small area, being restricted to high mountain ranges (Lefka Ori, 2452 m; Idi Oros, 2456 m; Dikti Oros, 2148 m), hills, and low plains of the island (8700 km^2^). The wide altitudinal range of this ecoregion results in several forest zones. The lowest elevations are distinguished by the predominance of sclerophyllous evergreen and semi-deciduous oak forests (*Quercus coccifera* L., *Quercus pubescens* Willd.), “maquis” of carob (*Ceratonia siliqua* L.), juniper (*Juniperus phoenicea* L.), and tree-spurge (*Euphorbia dendroides* L.). At medium altitudes, mesophyllous pine forests (*Pinus brutia* Ten.) and holly oak (*Quercus coccifera*) woodlands are widespread. The highest elevations host cypress (*Cupressus sempervirens* L.) woodlands, where maple trees (*Acer sempervirens* L.) frequently grow. In the high mountain elevations, extensive thorny cushion shrublands occur, which support many endemic species [31]. Crete is recognized by the International Union for Conservation of Nature as a “global centre of plant diversity” and is home to about 1800 species and subspecies of plants; its biodiversity is reflected not only in the total number of species, but also in the number of endemic species: 223 endemic species and subspecies, i.e., approximately 13% of the plant species of Crete [32].

In Crete, the impact of anthropogenic activities has been remarkable in some areas, mainly due to the over-use of pastures and grazing in mountain areas, growing tourism development, and some intensive agricultural activities in the southern coastal zone.

### 2.3. Brief Overview of Crete through Time

Crete is well known for having been the center of Europe’s most ancient civilization, the Minoans, which emerged by 3000 BC on the isle and other surrounding islands (Kea, Kythera, Milos, Rhodes, and Santorini). About 2000 BC, the Minoans had begun to build “palaces” on the sites of Knossós, Phaestus, and Mallia (Mália) [33]; the Minoan civilization was centered at Knossós and reached its peak in the 16th century BC, trading widely in the Eastern Mediterranean. The Minoans produced striking sculptures, frescoes, pottery, and metalwork. By about 1500 BC, Greek mainlanders from Mycenae assumed an influential role in Minoan affairs [33]. Minoans developed the Linear A writing system that was used to write the hypothesized Minoan language or languages: the script was written using a stylus to cut lines into a tablet of clay, as opposed to cuneiform, which was written by using a stylus to press wedges into the clay. After Crete suffered a major earthquake that destroyed Knossós and other centers around 1450 BC, power in the region passed decisively to the Mycenaeans, with whom Crete was closely associated until the commencement of the Iron Age around 1200 BC. About this time, the Dorians, another Greek-speaking people, moved southward and controlled the island. Crete played a supporting role in the revival of Greek civilization that began in the 9th century BC, and during Athens’s heyday in the 5th century BC, Crete fascinated the Greeks as a source of myths, legends, and laws. By 67 BC, the Romans appeared and completed their conquest of Crete, but in AD 395 the island passed to Byzantium (the Eastern Roman Empire); the Arabs gained control over parts of Crete after 824 but lost them back to the Byzantines in 961 [33]. In 1204, in the aftermath of the Fourth Crusade, crusaders sold the island to Venice, which incorporated Crete into its growing commercial empire. The Ottoman Turks, who were already in control of parts of Crete, wrested the capital city of Candia (now Heraklion) from the Venetians in 1669 after one of the longest sieges in history; Crete stagnated under Turkish rule, and native uprisings were always foiled, including those in 1821 and 1866. The Turks left in 1898, after which the island held autonomous status until its union with Greece in 1913 [33].

### 2.4. Current Ethnobotanical Field Study

The ethnobotanical field study was carried out in February 2022 in the study area and villages illustrated in Figure 1. The main purpose of the survey was to record local knowledge of wild greens (WGs) currently gathered and consumed by locals. Thirty-one study participants were recruited through snowball techniques to participate in semi-structured interviews, favoring middle-aged and elderly inhabitants (range: 52 to 78 years old), and especially rural farmers, shepherds, and elderly women, who were considered potential WGs local knowledge holders in the area. Additionally, the weekly Saturday farmer’s market in Heraklion and its daily vegetable markets were visited as well.

Prior to each interview, verbal consent was obtained from each of the participants and the Code of Ethics adopted by the International Society of Ethnobiology [34] was followed. Semi-structured interviews were conducted in Greek or English. For each of the WGs free listed during the study, the local name and local food uses were documented. We deliberately excluded from the survey wild seasoning plants (condiments) mainly used dried (i.e., wild oregano, wild thyme), as well as wild fruits, mushrooms, and wild snacks (i.e., wild plant parts ingested mainly for leisure outside food contexts/domestic arenas). The quoted wild food taxa were collected from the study area, when available, and identified by the first author using standard reference works concerning the Aegean flora [35,36]; identifications were later cross-checked with Cretan annotated checklists [37,38,39]. Voucher specimens (bearing numbers UVVETBOTCr01-38) were deposited at the Herbarium of the Bio-Cultural Diversity Lab of the Department of Environmental Sciences, Informatics and Statistics, Ca’ Foscari University of Venice, Italy. Identification of wild plants, which were not available during the field study, was conducted based upon the folk names and detailed plant descriptions; in this case, pictures of the presumed plants were shown to the study participants after a preliminary evaluation of the quoted folk name and description. Nomenclature always followed The World Flora Online database [40], while plant family assignments were consistent with the Angiosperm Phylogeny Website [41]. Recorded local Greek names were reported in Romanized transliterations following the rules approved by Hellenic Organization for Standardization, ELOT 743 standards [42] (ELOT); however, we rendered the voiceless postalveolar affricate tʃ with the sign č.

### 2.5. Data Analysis

A historical comparison was conducted in January–March 2022 analyzing the data gathered in the current study together with those reported by the German botanist Theodor Heinrich Hermann von Heldreich (1822–1902), who, after receiving botanical training in Montpellier (France) and Geneva (Switzerland), travelled extensively throughout Italy, Greece, Asia Minor, and Crete from 1843 to 1848, before eventually settling in Greece in 1851. There, he carried out botanical investigations, publishing thirteen volumes of the “Herbarium Graecum Normale” between 1856 and 1896, and served as director of the court garden, as well as director of the Natural History Museum. In 1862, he published a book *Die Nutzpflanzen Griechenlands mit besonderer Berücksichtigung der neugriechischen und pelasgischen Vulgarnamen* [27] on the useful plants of Greece, where both cultivated and wild plants widely used in Greece (mainly for medicine and food) were described.

Additionally, the data of the current study were compared with one of the very first ethnobotanical investigations ever carried out in the Mediterranean using ethnographic methods and specifically focusing on wild food plants, which was conducted on Corfu Isle by the archeologist Augustus Sordinas [28].

**Figure 4:**
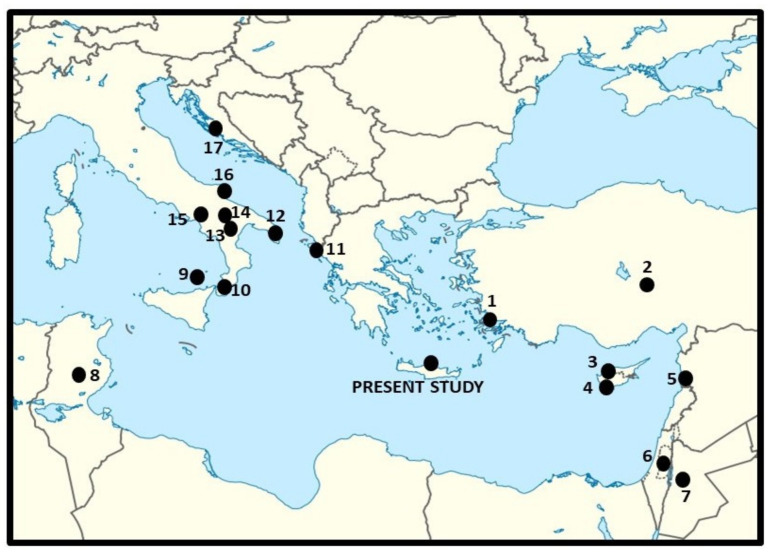
Mediterranean field study sites used in the comparative analysis; number and digits refer to the considered study sites listed in Table 1.

Moreover, the current data were compared with local food uses of WGs recorded in the past three decades during field investigations conducted in other Eastern and Central Mediterranean areas (Figure 4 and Table 1), i.e., Aegean Turkey [13,43,44], Cyprus [14,45], coastal Syria [46], Palestine [47], Western Jordan [48], Tunisia [49], Southern Italy [7,17,18,20,50], Greece [28], and Dalmatian Croatia [8]. We deliberately avoided considering national or large regional reviews on wild food plants, whose primary sources were unclear and/or possibly not based on genuine ethnography and modern ethnobiological methods, i.e., without any evidence of face-to-face interviews with locals and very sound documentation of local plant names.

Lastly, the data were compared with those we recorded in recent years among Assyrians in Iraqi Kurdistan [51], since most scholars tend to agree with *Assyrian continuity*, i.e., the theory of continuity between modern Assyrian people and the people of ancient Assyria, i.e., Mesopotamian Neolithic farmers [52].

## 3. Results and Discussion

### 3.1. Chorta Diversity and Their Traditional Food Uses in Central Crete

The wild-plant-based gastronomic heritage of central Crete includes fifty-five botanical taxa (Table 2).

Most of the recorded species are bitter Asteraceae (members of 13 genera), as also found one century ago by von Heldreich [27] in his Greek survey. Pungent Brassicaceae and aromatic Apiaceae are represented by 4 genera each. This is an important element, if we compare this picture with what has emerged from other surveys on WGs conducted in the Mediterranean (see following sections).

### 3.2. Folk Categorization of Cretan Chorta

In the study area, all study participants defined *Chorta* as wild greens; however, the cognitive categories underpinned in their folk classification were very unusual.

**Figure 5:**
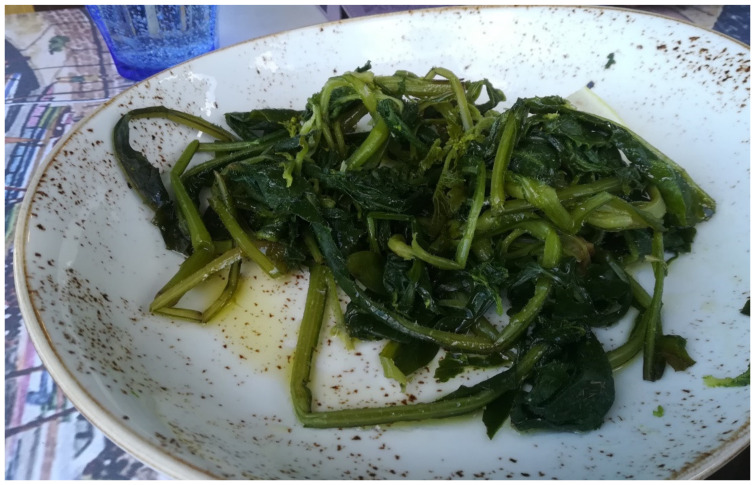
Boiled Cretan wild greens (*vrasta*).

Locals group *Chorta* according to their culinary processes and especially maintain the very popular category of *Vrasta* (wild greens consumed boiled with lemon and olive oil, Figure 5) and that of *Tsigariasta* or *Tsiagiasta* (wild greens fried in a pan and mainly later used as a filling for pies: phyllo-based pita—*chortopita*—and stuffed dough that is fried or baked—*kalitsounia*).

Moreover, there are other hidden categories (not lexicalized by a lexeme) referring to *Chorta* consumed raw in salads or prepared with eggs and meat. Within both the *Vrasta* and *Tsigariasta* categories, a distinction is made according to the taste: the most common *Vrasta* are indicated as “bitter” or sweet; the “bitter” cluster (including both bitter Asteraceae and pungent Brassicaceae) is culturally the most salient in the study area and study participants stressed the importance of the belief that these ingredients may support good health. The subgroup of *agrioradikio* includes the bitter Asteraceae, whose prototypes are represented mainly by wild *Cichorium*, *Taraxacum*, and *Crepis* spp. On the other hand, the most culturally salient and “spoken” *Chorta* for pita are those Apiaceae spp. considered to have an aromatic, pleasant taste, and which are deemed necessary for cooking a “proper” pie (i.e., *Scandix*, *Tordylium*, *Pimpinella*, *Foeniclum* spp.).

**Figure 6:**
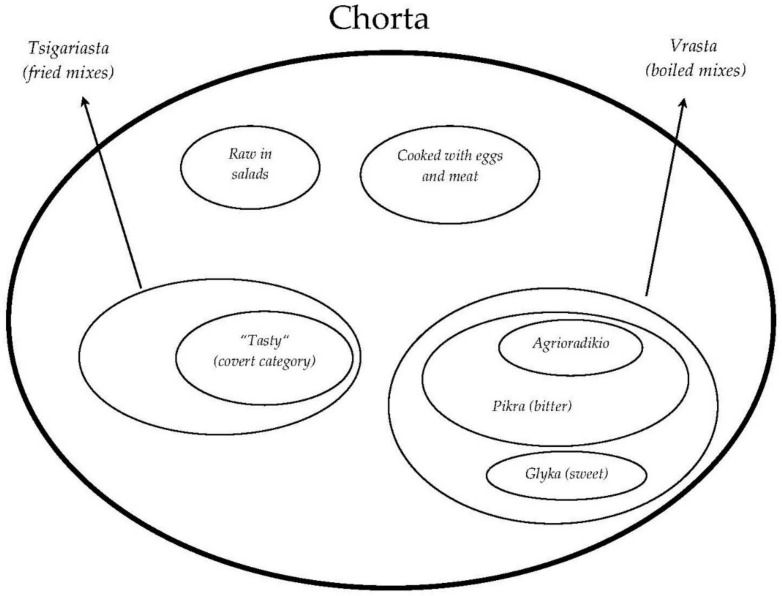
Folk classification of *Chorta* in the study area.

In Figure 6 we represent the folk categorization of *Chorta*, reporting in brackets some examples of prototypical botanical genera (see the following Table 2 for botanical details).

It is interesting to see how this assemblage is actually very similar to what Vulcano Isle (Northern Sicily) locals categorized for similar wild greens: *Minestra* (boiled wild greens consumed with lemon and oil) and *Minestra fritta* (boiled wild greens, later fried in a pan with olive oil, garlic, and tomatoes) [22].

Similarly, von Heldreich [27] classified the wild greens used by Greeks into three main categories: (a) *Lahana*—those wild greens, mainly bitter Asteraceae, which represent proper vegetables and the pillar of local sustenance (in turn grouped into those considered the best ones and those considered of secondary quality and only occasionally gathered); (b) wild greens predominately used raw as appetizers (mainly Brassicaceae); and (c) wild vegetable snacks, mainly consumed for leisure (green wild Fabaceae fruits, wild artichokes, garlic, *Crocus* spp. corms, *Scorozonera* roots, and young shoots of wild asparagus, *Dioscorea*, *Smilax*, and *Ruscus* spp.).

#### Cross-Historical and Cross-Cultural Comparisons

The recorded WV-centered ethnobotany of Crete was compared with the two aforementioned Greek historical sources [27,28], and the salient results are illustrated in Table 3. It is interesting to note some major changes in the past few decades: 1. Some archaic uses of hypogeal wild plant parts (for example, those of *Orchis* and *Cyperus* spp. tubercles and *Crocus* spp. corms) seem to have disappeared for historical reasons possibly linked to ecological changes (or increased awareness regarding the need to protect wild orchids) or to the decrease in the custom of snacking on raw plants (which were not, however, the focus of this survey); 2. The diversity of gathered pungent/bitter green Asteraceae and Brassicaceae seems to have decreased. This last aspect could be worthy of further investigation, since this could possibly be linked to the changes that the daily MD may have undergone in Crete during the past few decades, especially after industrialization and globalization of the food system, which have rendered the strong taste of some *Chorta* less appealing and appreciated.

In contrast, the cross-regional comparison (Table 4) shows that Cretan *Chorta* share the most similarities (mostly young aerial parts of Asteraceae and Brassicaceae) with the WGs gathered and consumed in (Greek speaking) Cyprus, the Bodrum area of Turkey, coastal Syria, and Southern Italy. This is quite pertinent since these common genera could be considered a possible bulk of an original post-Neolithic dietary system. The data collected in coastal Syria and in the Bodrum area of Turkey come in fact from territories that were heavily influenced by both autochthonous and Greek-Byzantine domination, while the pattern of ancient Greek influence is of course clearer for the Southern Italian data.

As suggested by [26] and further corroborated by our recent research in Mesopotamia [51], we also hypothesize that the main core of the Mediterranean WGs emerged during the Neolithic period and this is evident in the data we recently gathered among Assyrians in Mesopotamia (Iraqi Kurdistan), since Assyrians are considered the direct descendants of Mesopotamian Neolithic farmers [52]. This original bulk may have been preserved particularly well during the past two millennia among Greeks and Eastern Christians in the Levant, as well as in those Central Mediterranean areas more heavily influenced by both ancient Greek and Byzantine cultures. This could be due to the prominent role played by a strictly vegetarian diet during the fasting period in the Orthodox calendar and especially during the Lenten period. The influences of later and more lateral contributions to Mediterranean cultures (i.e., Arab, Turkish, Southern Slavic) may have diluted the “original” core (or enriched it with external elements; see data from sites 1 and 2 in Table 4), which is, however, still fairly well preserved in Dalmatia. The possible role that the Phoenicians may have played should also be considered, given the fact that Phoenician influence is still strong in coastal Syria, even leaving traces in the folk names of wild plants (i.e., *Rhus coriaria*, sumac, locally called “*the sour of the Phoenicians*” [46]).

Furthermore, the major predominance of WGs belonging to the bitter Asteraceae family over those belonging to the pungent Brassicaceae family, which clearly emerges from all the Near Eastern and Cretan data, and the contemporary increasing importance of Brassicaceae in the Sicilian dataset [18] may suggest a shift in the cultural appreciation of pungent tastes during the migration of the Neolithic WV-centered food heritage westwards.

**Figure 7:**
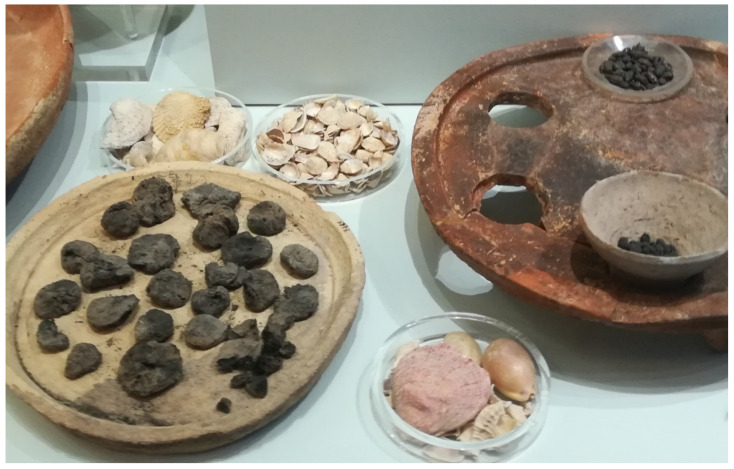
Carbonized Cretan figs, olives, and diverse shellfish, about 1500–1600 BC, Hagia Triada site, Heraklion Archaeological Museum.

If this is what the data suggest, it is of course advisable that further ethnobotanical research on the WGs of the MD should aim to better understand the movements of the wild plant portion of the post-Neolithic dietary systems based on cultivated cereals and pods, figs, olives, shellfish (Figure 7), and sheep and goat dairies from the Near East westwards into the Mediterranean. If it is in fact true that archaeobotanical studies cannot tell us much about leafy wild greens, thus making these ingredients “hidden” or invisible, a more systematic spectrum of ethnobotanical surveys specifically focusing on WGs could be crucial, especially favoring Eastern Christian diasporas and/or linguistic diasporas connected to old Levantine languages, such as that represented by the Assyrian language complex.

### 3.3. Wild Plant Mediterranean Food Heritage: Quo Vadis?

**Figure 8:**
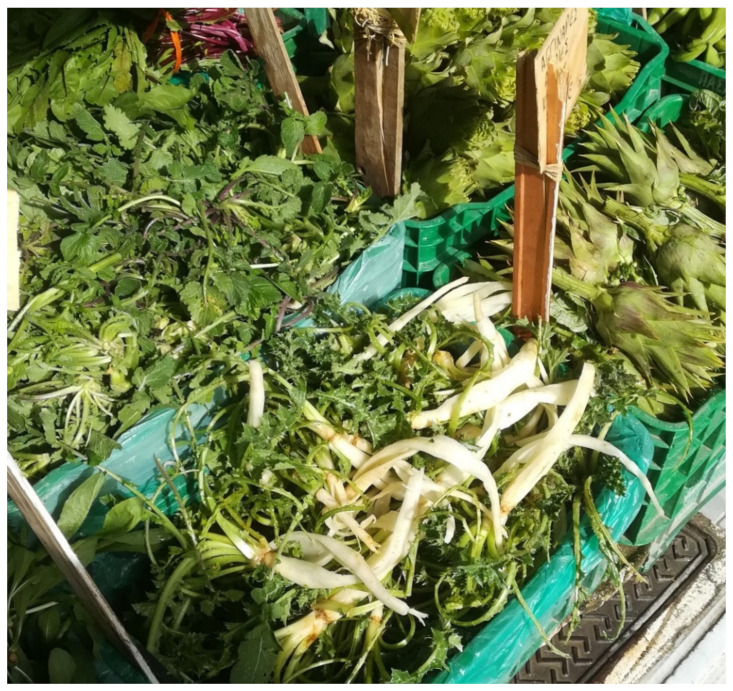
*Chorta* in a vegetable market in Heraklion.

The wild plant portion of the MD is not only a crucial part of local biological and cultural heritage but also an important generator of small-scale economies. Most of the recorded plants were also available in the local farmer’s market (Figure 8) and in Crete, *Chorta* are on the menu of almost every contemporary restaurant.

The resilience of *Chorta* heritage in Crete is remarkable and is assuredly linked primarily to the popularity this food still retains in the local population. Although “*young people do not know anything about Chorta*” (64 y.o. man), the study participants were keen to reaffirm the healthiness of these ingredients because they are also considered especially natural (“*we gather it in very pristine countryside areas*”—70 y.o. man).

A further factor which may have played a role in this resilience is the simple culinary processing of *Chorta* mixes (presenting mainly two preparations: boiled and fried). While in fact the traditional ecological knowledge (TEK) linked to identifying and gathering *Chorta* seems to be retained by both (middle-aged and elderly) men and women, their culinary processing is in the hands of women; since social changes in the Mediterranean have clearly affected the time spent in the kitchen [22], the simplicity of *Chorta* preparations is perhaps a factor which has compensated for the potential risk of these wild greens disappearing from Cretan tables. In order to maintain the practice of the use of *Chorta* circulating in society and especially among younger generations, well-designed educational programs are needed, such as introducing *Chorta* identification, gathering, and cooking classes into the school curriculum.

However, the fact that the TEK linked to *Chorta* is decreasing among younger generations may speak to its fate in the future; current observations seem to suggest that *Chorta* are still present in those households where elderly individuals continue to maintain ties to countryside work, while for others the availability of *Chorta* in the market represents a reasonable solution, thus consigning *Chorta* to the domain of traded goods/vegetables. Within this context, *Chorta* could also provide the backdrop for promoting a healthy lifestyle via local foods in sustainable tourist initiatives, at least during the spring. In this respect, a recent study also pointed out the important agro-food potential of neglected and underutilized plant species (NUS) in Crete, and, among them, a few wild edible greens [53].

Further studies and research will need to reveal more about the TEK distribution and diversity of traditional WGs of the MD, in both botanical and social terms.

## 4. Conclusions

This study recorded the WV-centered bio-cultural heritage of central Crete, which includes a variety of vegetables still frequently consumed, mainly boiled and fried. Comparison with historical ethnobotanical data showed remarkable resilience of this custom in the past century, while the cross-cultural/regional comparison suggested that *Chorta* may have developed from an original “bulk” of weedy vegetables used by Near Eastern Neolithic farmers, which are still very widespread in the Eastern Mediterranean and in those areas of the Central Mediterranean that had robust links to ancient Hellenic cultures. The current research may provide a baseline for future rural development, environmental, eco-touristic, and gastronomic programs aimed at further promoting the Mediterranean diet, and increasing the public’s awareness about the importance of keeping WGs in the daily diet. It is also advisable that further field studies be conducted with the aim of further assessing the spatial and temporal dynamics of the wild plant portion of the MD.

## Figures and Tables

**Table 1:** Details of the ethnobotanical field studies on wild greens used for the comparative analysis (see Figure 4).

Site Number	Area and Country	Ethnicity	Reference
1	Bodrum area, Turkey	Turks	[13]
2	Misti area, Turkey	Turks	[43], quoted in [44]
3	Leftke area, Northern Cyprus	Turks	[45]
4	Paphos and Larnaca areas, Cyprus	Greeks	[14]
5	Tartus area, Syria	Arabs	[46]
6	Palestine	Arabs	[47]
7	Western Jordan, Jordan	Arabs	[48]
8	Sidi Bouzid area, Tunisia	Arabs	[49]
9	Vulcano Isle, Italy	Italians	[22]
10	Graecanic Calabria area, Italy	Partially Italianized Greeks	[18]
11	Corfu Isle, Greece	Greeks	[28]
12	Salento area, Italy	Italianized Greeks	[20]
13	Dolomiti Lucane area, Italy	South Italians	[17]
14	Vulture area, Italy	Partially Italianized Albanians	[21]
15	Monti Picentini area, Italy	South Italians	[50]
16	Gargano area, Italy	South Italians	[7]
17	Dalmatia, Croatia	Croatians	[8]

**Table 2:** Recorded Cretan wild greens, their folk names, local culinary uses, occurrence in markets, and frequency of quotation.

Botanical Taxon or Taxa, Botanical Family; Voucher Specimen Code	Local Name(s)	Use Parts	Local Food Uses	Occurrence in Local Markets	Frequency of Quotation
***Allium** ampeloprasum* L., Amaryllidaceae; Cr01	Agriopraso	Whole plant	Fried in mixtures for pies or cooked together with potatoes	Yes	High
*Amarantus blitum* L. and possibly other *Amaranthus* spp., Amaranthaceae	Vlita	Young aerial parts	Boiled in mixtures	Yes	Medium
*Anchusa azurea* Mill., Boraginaceae	Agoglosi	Young aerial parts	Boiled in mixtures	No	Low
*Anthriscus sylvestris* (L.) Hoffm., Apiaceae; Cr14	Čirimidhia	Young aerial parts	Fried in mixtures as a filling for pies	Yes	Medium
*Asparagus aphyllus* subsp. *orientalis* (Baker) P.H. Davis, Asparagaceae; Cr33	Asfaragia, Sfaragas	Young shoots	Boiled alone or in omelets	Yes	Medium
*Asphodeline lutea* Rchb., Asphodelaceae; Cr06	Not recorded	Young aerial parts	Boiled in mixtures	Yes	Low
*Atractylis cancellata* L., Asteraceae	Saziči	Flower receptacles	Raw or boiled	No	Very low
*Beta vulgaris* L. subsp. *maritima*, Amaranthaceae; Cr03	Pazi, Lahana, Serpullo	Leaves	Alone, boiled, or in mixtures as a filling for pies; wrapping dolma	Yes	Low
*Borago officinalis* L., Boraginaceae, Cr92	Aporantsa	Young leaves	Raw in mixed salads with (cultivated) young shoots of fava beans and pea plants	No	Very low
*Capparis* spp., Capparaceae	Kapari	Flower buds and very young aerial parts	Pickled in mixed salads	Yes	Low
*Chenopodium album* L. and possibly other *Chenopodium* spp., Amaranthaceae	Agriospanako	Young leaves	Boiled in mixtures	No	Low
*Cichorium spinosum* L., Asteraceae; Cr32	Stamnagathi	Whorls	In mixed salads or boiled in mixtures	Yes	Medium
*Crepis commutata* (Spreng.) Greuter, Asteraceae; Cr13	Glikosirida	Rosettes	Boiled in mixtures	No	Low
***Crepis** vesicaria* L., Asteraceae and other bitter-tasting *Crepis and Cichorium* spp.; Cr10	Agrioradikio, Agrioradičo, Koknos	Rosettes	Boiled in mixtures	Yes	High
*Cynara cornigera* Lindl. and *Onopordum tauricum* Willd., Asteraceae; Cr08, Cr21	Agriaginara	Young stems and flower receptacles	Flower receptacles: raw in salads;Young stems: cooked, often together with lamb or goat meat	Yes	Medium
*Daucus carota* L. s.l., Apiaceae; Cr11	Stafilinikas, Xilera	Young aerial parts	Boiled in mixtures	Yes	Medium
*Dioscorea communis* (L.) Caddick & Wilkin, Dioscoreaceae	Avronies	Young shoots	Boiled alone or in omelets	No	Medium
*Diplotaxis viminea* (L.) DC., Brassicaceae; Cr27	Not recorded	Young shoots	Boiled in mixtures	Yes	Low
*Erodium cicutarium* (L.) L’Hér. and *E. moscatum* (Burm.f.) L’Hér., Geraniaceae; Cr22	Hoiromurides	Young aerial parts	Boiled in mixtures	Yes	Low
*Eruca vesicaria* (L.) Cav., Brassicaceae	Rocha	Leaves	Raw in mixed salads	No	Low
***Foeniculum** vulgare* Mill., Apiaceae; Cr09, Cr19	Maratho	Leaves	Fried in mixtures as a filling for pies or seasoning stewed potatoes and tomatoes	Yes	High
***Glebionis** coronaria* (L.) Cass. ex Spach, Asteraceae; Cr05	Agriamargarita	Young aerial parts	Raw in mixed salads or in boiled mixtures	Yes	Medium
***Glebionis** segetum* Fourr., Asteraceae; Cr69	Mandilida, Mantilida	Young aerial parts	Raw in mixed salads or in boiled mixtures	Yes	High
*Helminthotheca echioides* (L.) Holub, Asteraceae	Adres, Adrìa	Young rosettes	In mixtures, boiled	Yes	Low
***Hirschfeldia** incana* (L.) Lagr.-Foss., Asteraceae; Cr15	Pikrovruves, Prikovruvus	Young aerial parts	In mixtures, boiled or in pies	Yes	High
*Lactuca serriola* L., Asteraceae;	Pikralithra, Shiroburides, Shiromurides	Young leaves	In mixtures, boiled	No	Medium
*Leontodon tuberosus* L., Asteraceae; Cr23	Vočisa	Young leaves and roots	In mixtures, boiled	Yes	Low
***Leopoldia** comosa* (L.) Parl., Asparagaceae; Cr30	Askordulakos	Bulbs	Cooked in various ways; pickled	Yes	High
*Malva neglecta* Wallr., Malvaceae, Cr56	Molocha	Leaves	In boiled mixtures	No	Very low
*Mentha spicata* L., Lamiaceae; Cr37	Varzam, Varzamos	Young leaves	In mixtures in pies and in the filling for dolma	No	Low
*Oenanthe pimpinelloides* L., Apiaceae	Kurnupidi, Kurnopides, Kurnopodi	Young aerial parts	Fried in mixtures in pies	Yes	Medium
*Oxalis pre-caprae* L., Oxalidaceae, Cr78	Xinida	Leaves	Raw or in mixed salads	Yes	Very low
***Papaver** rhoeas* L., Papaveraceae; Cr20	Kutsunada, Paparuna	Young stems and leaves	In mixtures, boiled, or alone with goat or lamb meat	Yes	High
*Petromarula pinnata* A.DC., Campanulaceae; Cr23	Marulida	Young leaves	In salad mixtures or in boiled mixtures	Yes	Medium
*Phagnalon saxatile* (L.) Cass., Asteraceae; Cr24	Not recorded	Young leaves	In mixtures, boiled	Yes	Very low
***Pimpinella** cretica* Poir., *P. peregrina* L., Cr31 and ***Tordylium** apulum* L., Apiaceae; Cr02	Kafkalida, Karfalithra	Young aerial parts	In mixtures for pies	Yes	High
*Portulaca oleracea* L. aggr., Portulacaceae	Antrakla, Glistrida	Aerial parts	In mixed salads	Yes	High
***Prasium** majus* L., Lamiaceae; Cr17	Lagudohorto	Young shoots and leaves	In mixtures, boiled	Yes	High
*Ranunculus ficaria* L., Ranuncolaceae	Karakul	Young rosettes	In boiled mixtures	No	Very low
***Reichardia** picroides* (L.) Roth, Asteraceae; Cr18	Galatsida	Aerial parts	In mixed salads, or in mixtures, boiled	Yes	High
*Rumex acetosella* L. and possibly other acidic, *Rumex* spp., Polygonaceae	Xinidha	Leaves	In salad mixtures	No	Very low
*Rumex conglomeratus* Murray and possibly other, non-acidic, *Rumex* spp., Polygonaceae; Cr07	Lapatha, Lapatho	Leaves	In mixtures, boiled and in pies; as wrapping leaves for dolma	Yes	Medium
*Scabiosa atropurpurea* L., Dipsacaceae	Starovula	Young rosettes	Boiled in mixtures	No	Very low
***Scandix** pecten-veneris* L. and possibly *Scandix australis* L., Apiaceae; Cr12	Ahatsikas, Archardika, Mironi, Tsimullia	Young aerial parts	Fried in mixtures, as a filling for pies	Yes	High
***Scolymus hispanicus*** L., Asteraceae; Cr25	Askolimbros, Gules, Gulos, Skulosò	Young shoots, tender peduncles and rachis of leaves (sometimes with parts of the stems), underground part of the stems and external coats of the roots	Cooked alone with eggs and goat or lamb meat	Yes	High
*Silene vulgaris* (Moench) Garcke, Caryophyllaceae	Papules, Strufulia, Struvulia	Young shoots	In boiled mixtures	No	Low
*Sinapis arvensis* L., Brassicaceae; Cr16	Lapsanides	Young aerial parts	In mixtures, boiled	Yes	Medium
*Solanum nigrum* L. subsp. *nigrum*, Solanaceae	Stifnos	Leaves	In boiled mixtures	No	Medium
***Sonchus** oleraceus* L. and possibly other *Sonchus* spp., Asteraceae; Cr26	Zochia, Zochos	Young aerial parts	In boiled mixtures	Yes	High
*Taraxacum hellenicum* Dahlst. And possibly other *Taraxacum* spp., Asteraceae; Cr38	Kopana	Young rosettes	Boiled in mixtures	No	Low
*Urtica urens* L., Urticaceae	Artzinida, Tsuknides	Young leaves	Boiled in mixtures	No	Low
*Unidentified taxon*	Kalamači	Young leaves	In fried mixes		Low
*Unidentified taxon*	Saziči	Young leaves	Boiled in mixtures		Very low
*Unidentified taxon*	Achardiči	Young leaves	Boiled in mixtures		Very low
*Unidentified taxon*	Fillades	Young leaves	Boiled in mixtures		Very low

Frequency of quotation: high: quoted by 40–100% of the study participants; medium: quoted by 10–39% of the study participants; low: quoted by 2 or 3 study participants; very low: quoted by only one study participant. In bold we reported the most quoted botanical genera.

**Table 3:** Botanical genera of the Cretan wild greens also quoted as being consumed in Greece 50 years ago and during the 19th century.

Study	Area	Documented Botanical Genera Referring to Foraged Wild Greens
von Heldreich, 1862 [27]	All Greece (including Crete)	65 (30 in common): ***Allium***, ***Amaranthus***, ***Anchusa***, *Arthrocnemum*, ***Asparagus***, *Astragalus* *, ***Beta***, ***Borago***, *Brassica*, *Bunias*, *Campanula*, ***Capparis***, *Cardopatium*, *Carlina*, *Centaurea*, ***Chenopodium***, ***Cichorium***, *Crithmum*, ***Crepis***, *Crocus* *, ***Chrysanthemum***, ***Cynara***, *Cyperus* *, ***Dioscorea***, *Emex*, ***Eruca***, *Erucaria*, ***Foeniculum***, ***Hirschfeldia***, *Hyoseris*, *Hypochaeris*, *Lathyrus* *, ***Leontodon***, ***Leopoldia***, *Lotus* *, *Lycium*, *Nasturtium*, *Notobasis*, ***Malva***, ***Petromarula***, *Pistacia*, *Podospermum*, ***Portulaca***, ***Onopordum***, *Orchis*, ***Reichardia***, *Reseda*, *Roemeria*, ***Rumex***, *Ruscus*, *Salvia* *, ***Scandix***, *Scorzonera*, ***Scolymus***, ***Sinapis***, ***Silene***, ***Smilax***, ***Solanum***, ***Taraxacum***, *Tolpis*, ***Tordylium***, *Tragopogon*, *Urospermum*, ***Urtica***, *Vicia*
Sordinas, 1971 [28]	Corfu Isle	28 (20 in common): ***Allium***, ***Amaranthus***, ***Asparagus***, ***Capparis***, *Crocus* *, ***Cynara***, ***Daucus***, *Draba*, ***Eruca***, ***Foeniculum***, ***Helminthotheca***, ***Hirschfeldia***, ***Leopoldia***, ***Mentha***, *Melissa*, *Nasturtium*, *Orchis*, ***Papaver***, ***Portulaca***, *Raphanus*, ***Scandix***, ***Sinapis***, *Sisymbrium*, ***Solanum***, ***Sonchus***, ***Taraxacum***, ***Tordylium***, ***Urtica***

* Wild plant parts mainly consumed as occasional raw snacks and not within domestic arenas (not considered in the current study); genera that we recorded in present-day Crete are reported in bold.

**Table 4:** Comparison between Cretan wild greens (botanical genera) and those quoted in other ethnobotanical studies previously conducted in Eastern and Central Mediterranean and among Assyrians in Iraqi Kurdistan [51]; see Figure 3 and Table 2 for details; in bold we reported the areas where the similarities are higher.

Site Number	Area and Country	Inhabitants and Main Historical Influences	Research Years	Number of Interviews	Number of Botanical Genera Recorded as WGs (Excluding Snacks and Dried Wild Seasoning Plants)	Number of Botanical Genera in Common with Crete
1	**Bodrum area**, **Turkey**	Turks, but the area was heavily influenced over many centuries (until the 16th century) by Greek culture—Bodrum was known as Halicarnassus in antiquity	approx. 1999–2002	109	64	22
2	Misti area, Turkey	Currently Turks, but the area was inhabited by Greeks before 1924	1964	Not reported	22	9
3	Leftke area, Northern Cyprus	Turkish-speaking Cypriots	2013–2104	135	23	16
4	**Paphos and Larnaca areas**, **Cyprus**	Greek-speaking Cypriots	2003–2005	89	48	25
5	**Tartus area**, **Syria**	Autochthonous Arabs, but the area was influenced for many centuries by Phoenicians, Greeks, and Romans	2020–2021	50	45	23
6	Palestine	Autochthonous Arabs	2006	190	47	20
7	Western Jordan, Jordan	Autochthonous Arabs	approx. 1994–1997	Not reported	31	13
8	Sidi Bouzid area, Tunisia	Autochthonous Arabs	2014–2015	43	18	7
9	**Vulcano Isle**, **Italy**	Autochthonous Southern Italians originally coming from Northern Sicily	2016	Not reported	35	23
10	Graecanic Calabria area, Italy	Ancient Greek diaspora, nowadays heavily Italianized	2002–2003	36	25	17
11	**Corfu Isle**, **Greece**	Greeks, but the isle was heavily influenced by Venetian culture for a few centuries	approx. 1970	Not reported	31	23
12	**Salento area**, **Italy**	Ancient Greek diaspora, currently entirely Italianized	2016	30	32	17
13	**Dolomiti Lucane area**, **Italy**	Autochthonous Southern Italians	2002–2003	86	31	23
14	**Vulture area**, **Italy**	Albanian diaspora (moved to the area in the 16th century from the Greek Peloponnese)	2000–2001	62	36	22
15	**Monti Picentini area**, **Italy**	Autochthonous Southern Italians	2013–2015	64	51	22
16	**Gargano area**, **Italy**	Autochthonous Southern Italians	2011–2014	25	61	21
17	Dalmatia, Croatia	Croatians, but the area was ruled and inhabited by Venetians for centuries	2012	68	26	19
18	Nineveh Plain, Iraqi Kurdistan	Assyrians	2017	31	23	15

## Data Availability

All the data are provided in the article.
